# Red meat consumption, risk of incidence of cardiovascular disease and cardiovascular mortality, and the dose–response effect: Protocol for a systematic review and meta-analysis of longitudinal cohort studies

**DOI:** 10.1097/MD.0000000000017271

**Published:** 2019-09-20

**Authors:** Gidyenne Christine Bandeira Silva de Medeiros, Kesley Pablo Morais de Azevedo, Gabriella Xavier Barbalho Mesquita, Severina Carla Vieira Cunha Lima, David Franciole de Oliveira Silva, Isac Davidson Santiago Fernandes Pimenta, Ana Katherine da Silveira Gonçalves, Clélia de Oliveira Lyra, Grasiela Piuvezam

**Affiliations:** a Department of Nutrition; b Postgraduate Program in Public Health; c Postgraduate Program in Nutrition; d Postgraduate Program in Health Sciences; e Department of Public Health, Federal University of Rio Grande do Norte (UFRN), Natal, Brazil.

**Keywords:** cardiovascular disease, cardiovascular mortality, processed meat, protocol, red meat, unprocessed red meat

## Abstract

**Background:**

Red and (particularly) processed meats are high in cholesterol and saturated and solid fatty acids. Their consumption is considered one of the risk factors for metabolic disorders. Numerous studies demonstrated a possible association between red meat consumption and cardiovascular disease (CVD). In this protocol, we propose a systematic review of the literature to examine the associations of red meat consumption with CVD incidence and mortality, and explore the potential dose–response relationship.

**Methods:**

We will search MEDLINE/PubMed, Scopus, SciELO, LILACS, ScienceDirect, Web of Science, Cochrane (CENTRAL), WHOLIS, PAHO, and Embase. We will include prospective epidemiological studies (longitudinal cohort). Risk of bias will be assessed using the Newcastle-Ottawa scale (NOS). Four independent researchers will conduct all evaluations. Disagreements will be referred to a fifth reviewer. We will summarize our findings using a narrative approach and tables to describe the characteristics of the included studies. The heterogeneity between trial results will be evaluated using a standard chi-squared test with *P* < .05. We will conduct the study in accordance with the guideline of the Preferred Reporting Items for Systematic Review and Meta-analyses Protocols (PRISMA-P).

**Results:**

This review will evaluate the association between red meat consumption and incidence of CVD and mortality (primary outcome measures). The secondary outcome measure will include the dose–response effect.

**Conclusion:**

The findings of this systematic review will summarize the latest evidence of the association between red meat consumption and incidence of CVD and mortality and the dose–response effect through a systematic review and meta-analysis.

**Registration:**

PROSPERO CRD42019100914.

## Abstract 2

Supplemental Digital Content is available in the text

## 1 Introduction

Population eating patterns are changing rapidly in most countries. There is an emphasis on increased consumption of animal products, especially red meat, even in countries with large populations (such as China).^[1,2]^ These changes in food consumption promote reflections on population morbidity and mortality patterns and result in a transition in health conditions related to the frequency, magnitude, and distribution of diseases. There has been a global shift in the leading cause of mortality and morbidity from infectious to noncommunicable diseases.^[3]^ In this scenario, chronic noncommunicable diseases, including cardiovascular disease (CVD), cancer, diabetes, and chronic respiratory diseases, are the leading global cause of death and account for 70% of deaths worldwide.^[4]^ Major causes of chronic noncommunicable diseases include modifiable behavioral risk factors such as smoking, harmful alcohol consumption, physical inactivity, and improper diet.^[4]^ Dietary fat has been studied as a modifiable variable in the prevention and treatment of noncommunicable cardiometabolic disease.^[5]^

Recent evidence suggests that the Mediterranean dietary pattern, which features a diet rich in unsaturated fatty acids (healthy fats), may reduce cardiovascular events.^[5]^ In contrast, the consumption of cholesterol, saturated fatty acids, and sodium, all of which are present in red meat and, especially processed meat, are suggested as one of the risk factors for metabolic disorders.^[6]^ In this context, studies showed a possible association between red meat consumption and CVD.^[7–9]^ Hence, this systematic review and meta-analysis protocol aims to review the literature about the associations of red meat consumption with CVD incidence and mortality. It will also explore the dose–response relationship between meat consumption and CVD. The following review question will be considered: what is the association between red meat consumption and incidence of CVD and mortality considering the dose–response effect?

## 2 Methods

### 2.1 Study registration

We based the systematic review protocol on the Preferred Reporting Items for Systematic Reviews and Meta-Analyses Protocols (PRISMA-P) statement guidelines.^[10]^ This systematic review protocol has been registered on the PROSPERO database (CRD42019100914). Ethical approval is unnecessary.

### 2.2 Eligibility criteria

#### 2.2.1 Types of studies

We will include prospective epidemiological studies (longitudinal cohort) that have reported the associations of the consumption of unprocessed red meat and processed meat with the incidence of cardiovascular disease and mortality.

#### 2.2.2 Types of participants

We will include studies that follow up at red meat consumption in apparently healthy people.

#### 2.2.3 Exposure

We will include studies that evaluated the exposure the red meat (unprocessed red meat or processed meat) consumption and association with cardiovascular disease and mortality.

#### 2.2.4 Outcome measures

The primary outcome measures will be incidence of or mortality by CVD (coronary artery disease, stroke, heart failure, and other). The secondary outcome measures will include the effect of dose–response.

#### 2.2.5 Exclusion criteria

We will not include studies that: it was a study of animals; it was a study that the risk assessment only related to the consumption of nutrients (animal protein, fat, etc); it was a study that the risk assessment only related all meat (white meat, red meat, and processed meat). The results of the study were aggregated as total CVD, without any specific type of CVD (coronary artery disease, stroke, heart failure, or other).

### 2.3 Search methods for study identification

We will conduct comprehensive searches in 10 databases: MEDLINE/PubMed, Scopus, SciELO, LILACS, ScienceDirect, Web of Science, Cochrane Library, WHOLIS, PAHO, and Embase.

The search equation was defined considering the following items: diet as exposure; red meat consumption (unprocessed red meat and processed meat); cardiovascular diseases as an outcome; and the type of study, prospective epidemiological studies (longitudinal cohort) (Appendix 1). There will be no limitation of time and languages.

### 2.4 Study selection and data extraction.

Four reviewers will independently select and review titles and abstracts. Articles which meet the inclusion criteria will be included for full review. Any disagreement will be resolved by discussion with a fifth reviewer.

**Figure 1:**
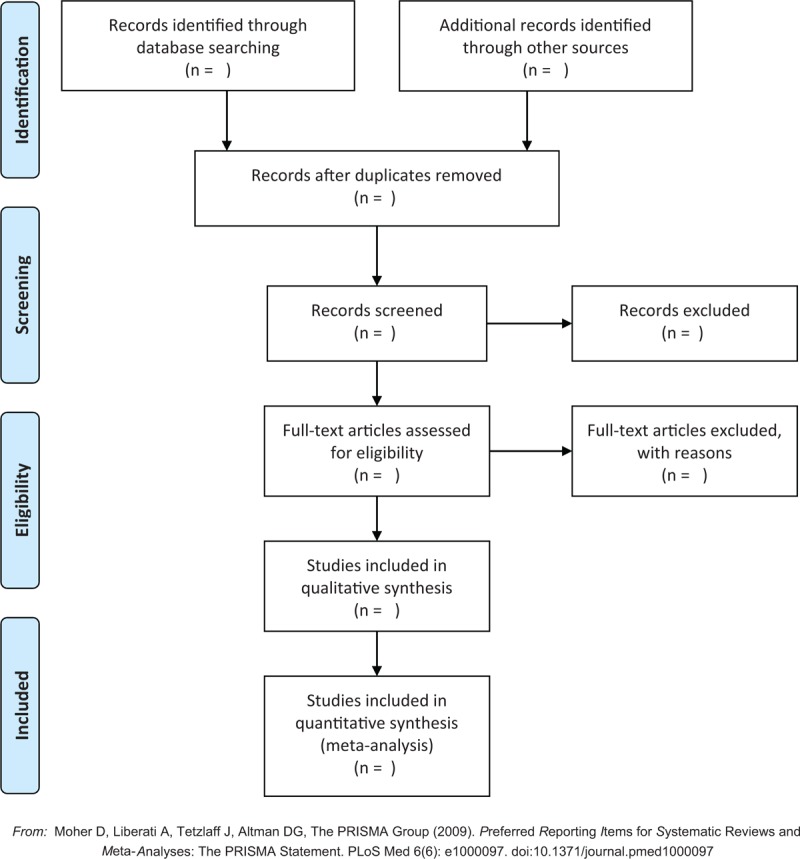
PRISMA flowchart of study selection. PRISMA = Preferred Reporting Items for Systematic Reviews and Meta-Analyzes.

The phases of the selection process will be identified in Fig. 1, based on the Preferred Reporting Items for Systematic Reviews and Meta-Analyzes (PRISMA).^[11]^

Two reviewers will extract the following information: title, first author, publication year, country, study name, population, participants characteristics (age, sex, sample size, no. events), duration of follow-up (y), person-years, disease outcome, disease ascertainment, dietary assessment, type of meat, consumption frequency or amount, statistical methods used for the analysis; risk estimates and confidence intervals, *P*-trend of dose–response test, and covariates that were matched or adjusted for in the multivariable analysis. When in doubt or discrepancies, a third researcher will be consulted.

### 2.5 Data analysis

#### 2.5.1 Risk of bias in the included studies

We will assess the methodological quality of the studies using the Newcastle-Ottawa scale (NOS).^[12]^ This scale evaluates the quality of non-randomized, cohort and case-control studies in relation to their design, content, and ease of use.

Four reviewers will independently carry out the evaluation. The disagreements will be resolved with a fifth reviewer.

#### 2.5.2 Statistical analysis

We will summarize by narrative approach and tables to describe the characteristics of the included studies.

The studies will structure around the type of CVD, characteristics of the target population, and consumption of different types of red meat (unprocessed red meat and processed meat). We will provide summaries the association of the end multivariate model of red meat consumption with CVD for each study according to sex, when the study does not present results by sex, we will present the results of the analysis with aggregated data.

When the study presents the risk analysis by red meat consumption ranges, we will provide a summary of the effects of the association between the highest consumption and the lowest.

The heterogeneity between trial results will be evaluated using a standard chi-square test with a significance level 0.05. We plan to compute the *I*^2^ statistic to assess heterogeneity. The *I*^2^ statistic is a quantitative measure of inconsistency across studies. *I*^2^ values of 50% indicate a substantial level of heterogeneity, whereas a value of 0% indicates no observed heterogeneity. Funnel plots will be used to assess the presence of potential reporting biases, if possible. A linear regression approach will be used to evaluate funnel plot asymmetry.

#### 2.5.3 Subgroup analyses

We will perform the following subgroup analyses by sex, type of red meat consumption, CVD sub types, and effect estimates according to their adjustments of covariates, if applicable.

## 3 Discussion

The proposed systematic review and meta-analysis will present studies that evaluated the associations of red meat consumption with CVD incidence and mortality, and the dose–response relationship between these factors. Current evidence suggests that increased meat consumption, especially red and processed meat, will negatively affect public health.^[2]^ According to the World Cancer Research Fund, there is strong evidence that consuming red and processed meat increases the risk of colorectal cancer.^[13]^

Studies demonstrated an association between red meat consumption with type 2 diabetes^[14]^ and CVD, such as coronary artery disease, stroke, and heart failure.^[8,14,15]^ A meta-analysis assessed dietary intake and risk of all-cause mortality and found that each additional daily intake of 100 g red meat is positively associated with the risk of all-cause mortality.^[16]^

Given the relevance of the evidence that indicates the consumption of red and processed meat as a risk factor for the development of chronic non-communicable disease^[8,14,15,17]^ and for all-cause mortality,^[16]^ it is opportune to investigate the association between this consumption and its gradient, as well as to relate it to CVD incidence and/or mortality, according to sex and disease type.

To estimate the long-term impacts of meat consumption, prospective epidemiological cohort studies are presented as the main approach, because participants are followed for years. Assessing the effect of red meat consumption through controlled clinical trials is extremely difficult to perform; it would require longer follow-up times to measure the long-term health effects. Additionally, experiments with nonhuman animals are difficult to interpret for relevance to human health.^[2]^

Therefore, the aim of the present study is to evaluate the association between red meat (unprocessed red and processed meat) consumption and CVD and mortality and the dose–response effect through a systematic review and meta-analysis of longitudinal cohort studies.

## Author contributions

**Conceptualization:** Gidyenne Christine Bandeira Silva de Medeiros, Gabriela Xavier Barbalho Mesquita, Grasiela Piuvezam.

**Data curation:** Gidyenne Christine Bandeira Silva de Medeiros, Kesley Pablo Morais de Azevedo, Clélia de Oliveira Lyra, Grasiela Piuvezam.

**Formal analysis:** Gidyenne Christine Bandeira Silva de Medeiros, Kesley Pablo Morais de Azevedo, Ana Katherine da Silveira Gonçalves, Grasiela Piuvezam.

**Investigation:** Gidyenne Christine Bandeira Silva de Medeiros, Kesley Pablo Morais de Azevedo, Gabriela Xavier Barbalho Mesquita, David Franciole de Oliveira Silva, Isac Davidson Santiago Fernandes Pimenta.

**Methodology:** Gidyenne Christine Bandeira Silva de Medeiros, Kesley Pablo Morais de Azevedo, Gabriela Xavier Barbalho Mesquita, David Franciole de Oliveira Silva, Isac Davidson Santiago Fernandes Pimenta, Grasiela Piuvezam.

**Project administration:** Gidyenne Christine Bandeira Silva de Medeiros, Severina Carla Vieira Cunha Lima, Clélia de Oliveira Lyra, Grasiela Piuvezam.

**Supervision:** Severina Carla Vieira Cunha Lima, Clélia de Oliveira Lyra, Grasiela Piuvezam.

**Writing – original draft:** Gidyenne Christine Bandeira Silva de Medeiros.

**Writing – review & editing:** Gidyenne Christine Bandeira Silva de Medeiros, Kesley Pablo Morais de Azevedo, Gabriela Xavier Barbalho Mesquita, David Franciole de Oliveira Silva, Isac Davidson Santiago Fernandes Pimenta, Ana Katherine da Silveira Gonçalves, Severina Carla Vieira Cunha Lima, Clélia de Oliveira Lyra, Grasiela Piuvezam.

Gidyenne Christine Bandeira Silva de Medeiros orcid: 0000-0001-5225-385X.

## Supplementary Material

Supplemental Digital Content
