# Supplementary material for: Red meat consumption, risk of incidence of cardiovascular disease and cardiovascular mortality, and the dose–response effect: Protocol for a systematic review and meta-analysis of longitudinal cohort studies
Source: Medicine (Baltimore). 2019 Sep 20;98(38):e17271. doi: 10.1097/MD.0000000000017271 (PMC6756738; doi:10.1097/MD.0000000000017271)
Supplement: Supplemental Digital Content [file medi-98-e17271-s001.docx]

**Appendix 1 -** Search strategies for MEDLINE/PUBMED database.

| **Database** | **Search strategies** |
| --- | --- |
| PubMed | (consumption OR intake) AND (meat OR “red meat” OR beef OR pork OR lamb OR goat OR “meat products” OR “processed meat” OR ham OR sausage OR hamburger OR bacon OR salami OR pastrami OR “luncheon meats”) AND (“cardiovascular disease” OR “coronary artery disease” OR “heart disease” OR “myocardial infarction” OR stroke OR “carotid artery disease” OR “heart failure” OR “heart attack” OR Atherosclerosis OR “cerebrovascular disease” OR “Peripheral vascular disease”) AND (cohort study) |
